# Tough on germs, gentle on hands: a cross-sectional study on hand skin health, skincare and hygiene habit changes among medical students in their clinical years in Dubai, the United Arab Emirates

**DOI:** 10.3389/fpubh.2025.1725837

**Published:** 2026-02-13

**Authors:** Zainab Al-Abdullah, Maryam Alabdullah, Fatma Alaghbari, Hamda Alfalasi, Amar Hassan, Aida Joseph Azar, Volha Shpadaruk

**Affiliations:** 1 Mohammed Bin Rashid University of Medicine and Health Sciences College of Medicine, Dubai, United Arab Emirates; 2 RENU Clinic, Dubai Healthcare City, Dubai, United Arab Emirates

**Keywords:** allergic dermatitis, hand skin health, eczema, hand dermatitis, moisturizer, occupational hand dermatitis, medical students, hand skincare

## Abstract

**Introduction:**

Occupational hand dermatitis (OHD) has become increasingly prevalent in recent years among healthcare worker populations worldwide. The condition can be so debilitating that it leads to sick leave or even career changes. This cross-sectional study aimed to investigate the impact of hand hygiene practices on hand skin health and skin care habits among undergraduate medical students in their clinical years, who are preparing to enter the healthcare workforce.

**Methods:**

The study population included undergraduate Doctor of Medicine (MD) program students who have completed at least 1 year of clinical rotations. Data was collected through an anonymous survey distributed to eligible students, with data collected from their pre-clinical and clinical years.

**Results:**

A total of 81 students completed the survey. In pre-clinical years, mean daily handwashing and sanitiser use were 6.1 and 4.6 times, respectively, with most (74%) spending < 5 min on hand hygiene. In clinical years, 63% of students spent >5 min daily on hand hygiene, 93% reported increased handwashing, and 52% noted worsened hand skin health. More females than males reported worsening of hand skin health during specific clinical placements (*p* = 0.001). WHO protocol adherence also improved significantly, with 51.9% of students being “always” compliant in clinical vs. 22.2% in pre-clinical years (*p* = 0.0001).

**Conclusion:**

Clinical training was associated with increased hand hygiene adherence but also a significant worsening of hand skin health, particularly among female students. High-contact rotations were most frequently linked to symptom exacerbation, highlighting the need for targeted skin protection measures during clinical years and in the healthcare environment.

## Introduction

Occupational hand dermatitis (OHD) is an umbrella term encompassing irritant and allergic contact dermatitis resulting from workplace exposures (1). It represents the most common occupational skin disease, with healthcare workers being among the highest-risk groups due to frequent handwashing, exposure to irritants, and prolonged glove use (1, 2). Known risk factors for OHD include a history of atopic dermatitis, frequent hand hygiene practices, contact with bodily fluids, and occlusive glove use (1, 3–5).

The burden of OHD has increased further following the COVID-19 pandemic, with multiple studies reporting a higher prevalence of hand dermatitis and related symptoms among healthcare workers worldwide (6–8). Beyond its dermatologic manifestations, OHD can become a burden, leading to reduced work performance, sick leave, psychological distress, and in some cases, career modification or discontinuation (2, 3).

Appropriate hand skincare practices, including the use of moisturizers and barrier-protective agents, have shown to reduce the severity and impact of OHD and support adherence to hand hygiene protocols in clinical settings (3, 9–11). However, despite the global recognition of OHD as an occupational health concern, data on its prevalence, risk factors, and hand skincare practices among healthcare workers in the United Arab Emirates (UAE) remain limited.

To date, no studies have specifically examined OHD among medical students in the UAE or the wider Gulf region. As medical students transition into clinical environments with increasing exposure to infection control measures, understanding the prevalence of hand dermatitis and its impact on hand hygiene behavior is essential. This study therefore aims to investigate hand skin health, hand hygiene practices, and associated factors among medical students during their clinical years in the UAE, to inform preventive strategies in training and healthcare settings.

## Materials and methods

### Study design

This cross-sectional study examined the impact of hand hygiene habits on hand skin health among medical students in their clinical years. Data collected via an anonymous English-language survey on Microsoft Forms distributed to students via a link, and it remained accessible for 3 months (kindly refer to Supplementary material). Students were permitted to respond during this period, and informal reminders were communicated through word of mouth. No standardized reminder schedule was used.

The questionnaire consisted of both adapted and newly developed items. A limited number of questions assessing the presence, symptoms, and healthcare-seeking behavior related to occupational hand eczema were asked using previously published surveys in healthcare workers (12, 13) and then reworded to suit the medical student population and local clinical training context. The majority of the questionnaire was developed specifically for this study to capture current hand hygiene practices, the presence of hand skin conditions, and perceived changes in hand skin health during medical training. Information on these variables was collected for both the pre-clinical and clinical years to allow for direct comparison of changes over time. Questionnaire development was guided by the study objectives and supervised by a consultant dermatologist. The questionnaire was used to describe students' experiences and perceptions and was not intended to establish diagnoses; therefore, formal questionnaire validation was not performed.

### Ethical approval

Ethical approval was obtained from the MBRU-Institutional Review Board prior to the commencement of the study (Reference #MBRU IRB-2024-543) and by the Dubai Scientific Research Ethics Committee (DSREC), Dubai Health Authority (Reference #DSREC-06/2025_21). Participants provided informed consent through the first page of the electronic survey link that contained an English information page before the commencement of the study.

### Study setting and participants

The population included all students who had completed at least 1 year of clinical rotations enrolled in the Doctor of Medicine (MD) program at the College of Medicine (CoM), Mohammed Bin Rashid University of Medicine and Health Sciences (MBRU), and the most recent graduating cohort from MBRU. The MD program is a 6-year undergraduate program, with the first 3 pre-clinical years (Years 1–3) focusing on building the medical foundation necessary to progress to the next stage. The last 3 years (Years 4–6) centers on hands-on clinical clerkship training across a diverse healthcare landscape in the UAE. During their clerkship training, students rotate full-time in public and private healthcare facilities. Expected to be active members of the healthcare team, they need to comply with hand hygiene standards set by local health authorities and infection control teams.

Pre-clinical students and clinical students who have not experienced 1 year of clinical rotations were excluded, as their experiences were insufficient to significantly impact their skin health. Likewise, more senior MBRU alumni were excluded to minimize recall bias.

### Study variables

Demographic data collected included year of study or graduation, gender, age, nationality, family history of chronic skin conditions, and use of nail varnish. Additionally, data related to pre-clinical versus clinical years included any personal history of chronic skin conditions and whether it specifically affected their hands, history of dermatologist or other doctor visits, skincare routine, as well as frequency of sanitizer usage and handwashing with soap using the World Health Organization (WHO) handwashing protocol (14). For this study, the term “chronic skin disease” was intentionally left broad and undefined to self-report any ongoing or recurrent skin conditions they personally perceived as chronic. This approach was chosen to capture the subjective burden of skin disease, rather than to impose strict diagnostic or duration-based criteria. Participants were therefore free to report any condition they considered chronic, regardless of formal diagnosis or specific chronicity thresholds. From an investigator's perspective, this category was intended to encompass common chronic dermatologic conditions such as hand eczema, atopic dermatitis, psoriasis, and other persistent inflammatory skin disorders; however, responses were accepted based on participant interpretation alone. No objective diagnostic verification or pre-defined chronicity criteria were applied.

Further data was collected on personal observations of changes in hand skin health compared to pre-clinical years and alterations in hand skincare routines after entering the clinical environment. Moreover, data were collected on variations in handwashing frequency, observations in hand skin health based on specialty placement, frequency of glove usage, and the effect of poor skin health on compliance with hand hygiene protocols. Finally, data were collected on students' perceptions of the effectiveness of different hand hygiene methods as well as their effect on hand skin health.

### Statistical methods

Categorical data were summarized using frequency and proportion, while continuous variables were described using measures of central tendency and dispersion. The Kolmogorov–Smirnov test was used to assess the normality of continuous data. Depending on the data distribution, either an independent samples *t*-test was used to compare continuous variables, or the Mann–Whitney *U* test was applied when the data were not normally distributed. Categorical data were analyzed using the Chi-square test or Fisher's exact test, as appropriate. All data analysis was performed using IBM SPSS Statistics for Windows, version 29.0 (IBM Corp., Armonk, NY, USA). A *p*-value of less than 0.05 was considered statistically significant.

## Results

A total of 81 undergraduate medical students out of 149, across three cohorts (Classes of 2024 to 2026) completed the survey (81/149; Table 1), yielding a response rate of 54.4%. This rate exceeded the average online survey response rate of 44% reported in a meta-analysis by Wu et al. (15), thereby supporting the adequacy of our sample size. The majority of respondents were female carrying 77.8% of the study (63/81), and the mean age (standard deviation) of the sample was 23.1 years (1.85), with no gender-based statistical significance difference. UAE nationals comprised 29 students of the sample standing at 35.8% (29/52), which was reflective of the university's population. A significantly higher proportion of female students 58.7% (37/41) reported a family history of chronic disease compared to male students of 22.2% (4/41; *p* = 0.006). Eczema (atopic dermatitis) was the most reported family history and was more common among females (33 vs. 4). Other skin conditions such as allergic contact dermatitis and psoriasis were also more frequently reported by females (Table 1).

**Table 1:** Demographic characteristics and family history stratified by gender.

**Demographic**	**Male** **No. (%)** ***N* = 18**	**Female** **No. (%)** ***N* = 63**	**Total** **No. (%)** **(*N* = 81)**	***p*-Value**
**Cohort**				
2024	1 (5.6)	20 (31.7)	21 (25.9)	0.081
2025	8 (44.4)	21 (33.3)	29 (35.8)	
2026	9 (50.0)	22 (34.9)	31 (38.3)	
Age (years), mean (SD) [range]	22.5 (1.125) [21–25]	23.3 (1.95) [21–34]	23.1 (1.85) [21–34]	0.116
**Nationality**				
UAE National	2 (11.1)	27 (42.9)	29 (35.8)	0.011
UAE non-National	16 (88.9)	36 (57.1)	52 (64.2)	
**Family history of chronic disease** ^1^				
No	14 (77.8)	26 (41.3)	40 (49.4)	0.006
Yes	4 (22.2)	37 (58.7)	41 (50.6)	
*Eczema (atopic dermatitis)*	4	33	37	
*Allergic contact dermatitis*	0	8	8		
*Irritant contact dermatitis*	0	1	1	
*Psoriasis*	1	6	7	
**Latex allergy**				
No	18 (100)	61 (96.8)	79 (97.5)	0.603
Yes	0 (0)	2 (3.2)	2 (2.5)	
**Regular use of nail varnish**				
No	17 (94.4)	51 (81)	68 (84.0)	0.156
Yes	1 (5.6)	12 (19.0)	13 (16.0)	
*Normal nail polish*	1	8	9 (11.0)	
*Gel Polish*	0	1	1 (1.2)	
*Hard gel*	0	2	2 (2.5)	
*Acrylic nails*	0	1	1 (1.2)	

N, number of participants; No., number; SD, standard deviation; UAE, United Arab Emirates.

^1^More than one response possible.

Among medical students in their pre-clinical years, eczema was the most reported chronic skin disease with a higher frequency in females (Table 2). Female students were also more likely to report having a skincare routine and use skincare products, including prescription topicals, non-prescription creams, and hand sanitisers compared to their male counterparts. The daily mean (standard deviation) handwashing frequency was 6.1 (3.2) and hand sanitiser use was 4.6 (4.7) for both males and females. There was no statistical significance found between male and female pre-clinical students regarding reported chronic disease, frequency of handwashing with soap and water or hand sanitiser, symptoms of poor hand skin health (itching, drying, burning or fissuring) and self-reported adherence to hand hygiene protocols (Table 2).

**Table 2:** Hand skin health and skincare practices among medical students in preclinical years (Years 1 to 3) stratified by gender.

**Question**	**Male** **No. (%)** **(*N* = 18)**	**Female** **No. (%)** **(*N* = 63)**	**Total** **No. (%)** **(*N* = 81)**	***p*-Value**
**History of chronic disease**				
No	13 (72.2)	35 (55.6)	48 (39.3)	0.159
Yes^1^	5 (27.8)	28 (44.4)	33 (40.7)	
*Eczema (atopic dermatitis)*	3	15	18	
*Allergic contact dermatitis*	0	6	6	
*Irritant contact dermatitis*	0	5	5	
*Psoriasis*	0	3	3	
*Other*	2	1	3	
**Hand skin health**				
Symptoms affecting hand skin				
No	16 (88.9)	51 (81.0)	67 (82.7)	0.348
Yes^1^	2 (11.1)	12 (19.0)	14 (17.3)	
*Itching*	1	11	12	
*Burning, pain, tenderness*	1	5	6	
*Dryness, tightnes*s	1	11	12	
*Fissuring*	0	4	4	
*Blisters, vesicles*	0	2	2	
*Inability to perform activities*	0	0	0	
Visit to a dermatologist				
No	17 (94.4)	58 (92.1)	75 (92.6)	0.733
Yes	1 (5.6)	5 (7.9)	6 (7.4)	
**Hand skincare routine**				
No	15 (83.3)	34 (54.0)	49 (60.5)	0.025
Yes^1^	3 (16.7)	29 (46.0)	32 (39.5)	
*Prescription topical medications*	1	3	4	
*Non-prescription petroleum cream*	1	1	2	
*Non-prescription ceramide*	0	2	2	
*Non-prescription urea*	1	3	4	
*Non-prescription, unsure*	1	11	12	
*Hand sanitizer*	2	14	16	
*Oral medication*	0	1	1	
**Hand hygiene**				
Number of daily soap-water handwashes, mean (SD) [range]	6.1 (4.3) [2–20]	6.1 (2.9) [2–15]	6.1 (3.2) [2–20]	0.457
Number of daily hand sanitizer uses, Mean (SD) [range]	4.6 (4.6) [0–20]	4.7 (4.8) [0–20]	4.6 (4.7) [0–20]	0.735
Adherence to WHO hand hygiene protocol	2.4 (1.4) [1–5]	2.9 (1.5) [1–5]	2.9 (1.5) [1–5]	0.168
*Never*	5 (27.8)	13 (20.6)	18 (22.2)	0.369
*Rarely*	7 (28.9)	15 (23.8)	22 (27.2)	
*Sometimes*	1 (5.6)	11 (17.5)	12 (14.8)	
*Often*	3 (16.7)	8 (12.7)	11 (13.6)	
*Always*	2 (11.1)	16 (25.5)	18 (22.2)	
Total daily time spent performing WHO hand hygiene protocol				
Less than 5 min/day	12 (66.7)	48 (76.2)	60 (74.1)	0.299
More than 5 min/day	6 (33.3)	15 (23.8)	21 (25.9)	

N, number of participants; No., number; %, percentage; SD, standard deviation.

^1^More than one response possible.

Worsening of hand skin health increased once medical students entered their clinical years, with this being most common amongst female respondents (*n* = 38, 60.3%), while males (*n* = 12, 66.7%) reported no change in their hand skin health, and very few respondents noticed improvement in their hand skin health in their clinical years (*p* = 0.007; Table 3). Females (39, 61%) were significantly more likely to have altered their skincare routine during the clinical years compared to males (*p* = 0.03), with higher use of both prescription and non-prescription products. There was also a trend toward significance in hand hygiene adherence and time spent (both *p* = 0.06), with females more likely to report following protocols and spending over 5 minutes handwashing daily (Table 3). Most clinical students (75, 92.6%) reported an increase in handwashing frequency during clinical years, especially during high-contact placements. Worsening of hand skin health during specific clinical placements was reported by 29 (46.0%) females and 1 (5.6%) male (*p* = 0.001), most commonly during surgery (*n* = 25), medicine (*n* = 19), emergency medicine (*n* = 12) and ICU/NICU (*n* = 16), mainly reported by female students (Table 3). Similar to pre-clinical students, reported chronic skin disease, frequency of handwashing with soap and water or hand sanitiser, in addition to usage of gloves, were also not shown to be statistically significant between male and female during their clinical years (Table 3).

**Table 3:** Hand skin health and skincare practices among medical students in clinical years (Years 4 to 6) stratified by gender.

**Question**	**Male** **No. (%)** **(*N* = 18)**	**Female** **No. (%)** **(*N* = 63)**	**Total** **No. (%)** **(*N* = 81)**	***p*-Value**
**History of chronic disease**				
No	13 (72.2)	35 (55.5)	48 (59.3)	0.159
Yes^1^	5 (27.8)	28 (44.5)	33 (40.7)	
*Eczema (atopic dermatitis)*	3	15	33	
*Allergic contact dermatitis*	0	6	6	
*Irritant contact dermatitis*	0	5	5	
*Psoriasis*	0	1	1	
*Other*	2	1	3	
**Hand skin health**				
Symptoms affecting hand skin				
No	16 (88.9)	44 (69.8)	60 (74.1)	0.089
Yes^1^	2 (11.1)	19 (30.2)	21 (25.0)	
*Itching*	1	14	15	
*Burning, pain, tenderness*	1	9	10	
*Dryness, tightness*	1	18	19	
*Fissuring*	0	0	0	
*Blisters, vesicles*	0	1	1	
*Inability to perform daily activities*	0	2	2	
Visit to dermatologist				
No	18 (100)	60 (95.2)	78	1
Yes	0 (0)	3 (4.7)	3	
Overall hand skin health in clinical years compared to pre-clinical years				
No difference	12 (66.7)	24 (38.1)	36 (44.4)	0.007
Hand skin improved	2 (11.1)	1 (1.6)	3 (3.7)	
Hand skin worsened	4 (22.2)	38 (60.3)	42 (51.9)	
**Hand skincare routine**				
No	12 (66.7)	24 (38.1)	36 (44.4)	0.089
Yes^1^	6 (33.3)	39 (61.9)	45 (55.6)	
*Prescription topical medication*	1	6	7	
*Non-prescription petroleum cream*	2	9	11	
*Non-prescription ceramide*	1	15	16	
*Non-prescription urea-based cream*	0	6	6	
*Non-prescription (unsure of type)*	0	9	9	
*Hand sanitizer*	2	16	18	
*Oral medication*	0	2	2	
**Hand hygiene**				
Number of daily soap-water handwashes, Mean (SD) [range]	7.9 (6.1) [1–20]	9.8 (7.4) [2–50]	9.4 (7.2) [1–50]	0.169
Number of daily hand sanitizer uses, Mean (SD) [range]	8.3 (5.7) [1–20]	10.7 (8.8) [0–5]	10.1 (8.2) [0–50]	0.329
Adherence to WHO hand hygiene protocol	3.6 (1.3) [1–5]	4.3 (0.9) [1–5]	4.2 (1.1) [1–5]	0.014
*Never*	1 (5.6)	1 (1.6)	2 (2.5)	0.06
*Rarely*	3 (16.7)	2 (3.2)	5 (6.2)	
*Sometimes*	5 (27.8)	8 (12.7)	13 (16.0)	
*Often*	3 (16.7)	16 (25.4)	19 (23.5)	
*Always*	6 (33.3)	36 (57.1)	42 (51.9)	
Total daily time spent performing WHO hand hygiene protocol				
Less than 5 min/day	10 (55.6)	20 (31.7)	30 (37.0)	0.06
More than 5 min/day	8 (44.4)	43 (68.3)	51 (63.0)	
Daily instances of glove use, Mean (SD) [range]	4.3 (2.3) [1–10]	6.2 (4.5) [0–25]	5.8 (4.2) [0–25]	0.114
Poor hand skin health negatively affecting adherence to WHO hand hygiene protocol				
No	15 (83.3)	52 (82.5)	67 (82.7)	0.623
Yes^1^	3 (16.7)	11 (17.5)	14 (17.3)	
*Wearing gloves to avoid hand washing*	0	8	8	
*Refraining from hands-on contact with patients*	1	5	6	
*Rushing through hand cleaning*	2	2	4	
*Not covering the entire hand during washing*	0	3	3	
*Avoiding soap*	1	1	2	
*Avoiding hand sanitizer*	3	4	7	
**Effect of specialty placement**				
Increased handwashing				
No	3 (16.7)	3 (4.8)	6 (7.4)	0.12
Yes^1^	15 (83.3)	60 (95.2)	75 (92.6)	
*Surgery*	14	46	60	
*Medicine (including-sub-specialities)*	12	38	50	
*Pediatrics*	12	44	56	
*Family Medicine*	2	20	22	
*Obstetrics & Gynecology*	3	38	41	
*Psychiatry*	1	5	6	
*Emergency medicine*	7	37	34	
*ICU/NICU*	8	42	50	
Worsened skin health				
No	17 (94.4)	34 (54.0)	51 (62.9)	0.001
Yes^1^	1 (5.6)	29 (46.0)	30 (37)	
*Surgery*	0	25	25	
*Medicine (including-sub-specialities)*	1	18	19	
*Pediatrics*	1	14	15	
*Family Medicine*	0	8	8	
*Obstetrics & gynecology*	0	10	10	
*Psychiatry*	0	3	3	
*Emergency medicine*	0	12	12	
*ICU/NICU*	0	16	16	

N, number of participants; No., number; %, percentage; SD, standard deviation; ICU, intensive care unit; NICU, neonatal intensive care unit.

^1^More than one response possible.

Some clinical students (*n* = 14, 17.3%) reported that poor hand skin health affected hand hygiene, often by rushing, avoiding soap/sanitiser, or using gloves (Table 3).

Compared to students in their pre-clinical years, clinical students were more likely to experience symptoms of poor hand skin health (25 vs. 17.3%) and experience symptoms of poor skin health such as itching, burning, and dryness (Table 4). Skincare routines were also significantly more common during clinical years compared to pre-clinical years (55.6 vs. 39.5%; *p* = 0.041) with increased use of prescription topicals, non-prescription moisturizers, and ceramide-based creams. Clinical students were also statistically significantly more likely to adhere to hand hygiene protocols (51.9 vs. 22.2%; *p* = 0.0001) and spend more time on hand hygiene (63 vs. 25.9%; *p* = 0.0001) compared to pre-clinical students (Table 4). Those in their clinical years also had significantly more frequent hand hygiene practices in comparison to those in their pre-clinical years, with a higher mean number of daily hand washes (9.4 vs. 6.1; *p* = 0.0002) and hand sanitiser uses (10.1 vs. 4.6; *p* = 0.0001). The proportion of students with a chronic disease history remained the same across both groups (40.7%) while visits to a dermatologist were low among both groups (Table 4).

**Table 4:** Comparison of hand hygiene practices and hand skin health among undergraduate medical students in pre-clinical (Years 1–3) and clinical (Years 4–6) years.

**Compared variable**	**Pre-clinical No. (%) (*N* = 81)**	**Clinical No. (%) (*N* = 81)**	***p*-Value**
**History of chronic disease** ^1^			
No	48 (59.3)	48 (59.3)	0.207
Yes^1^	33 (40.7)	33 (40.7)	
*Eczema (atopic dermatitis)*	18	18	
*Allergic contact dermatitis*	6	6	
*Irritant contact dermatitis*	5	1	
*Psoriasis*	3	5	
*Other*	3	3	
**Chronic conditions affecting hand skin**			
No	67 (82.7)	60 (74.1)	0.181
Yes^1^	14 (17.3)	21 (25.0)	
*Itching*	12	15	
*Burning*	6	10	
*Dryness*	12	19	
*Fissuring*	4	0	
*Blisters*	2	1	
*Inability to perform activities*	0	2	
**Visit to dermatologist**			
No	75 (92.6)	78 (96.3)	0.303
Yes	6 (7.4)	3 (3.7)	
**Skin care routine**			
No	49 (60.5)	36 (44.4)	0.041
Yes^1^	32 (39.5)	45 (55.6)	
*Prescription topical medications*	4	7	
*Non-prescription petroleum cream*	2	11	
*Non-prescription ceramide*	2	16	
*Non-prescription urea*	4	6	
*Non-prescription, unsure*	12	9	
*Hand sanitizer*	16	18	
*Oral medication*	1	2	
**Hand hygiene**			
Number of daily soap-water handwashes, Mean (SD) [range]	6.1 (3.2) [2–20]	9.4 (7.2) [1–50]	0.0002
Number of hand sanitizer uses per day, Mean (SD) [range]	4.6 (4.7) [0–20]	10.1 (8.2) [0–50]	0.0001
Adherence to WHO hand hygiene protocol	2.9 (1.5) [1–5]	4.2 (1.1) [1–5]	0.0001
*Never*	18 (22.2)	2 (2.5)	0.0001
*Rarely*	22 (27.2)	5 (6.2)	
*Sometimes*	12 (14.8)	13 (16.0)	
*Often*	11 (13.6)	19 (23.5)	
*Always*	18 (22.2)	42 (51.9)	
Total daily time spent performing WHO hand hygiene protocol			
Less than 5 min/day	60 (74.1)	30 (37.0)	0.0001
More than 5 min/day	21 (25.9)	51 (63.0)	

N, number of participants; No., number; %, percentage; SD, standard deviation.

^1^More than one response possible.

Additionally, 58 (71.6%) of students rated soap as more effective and gentler than sanitiser and 75 (92.6%) viewed gloves as essential for infection control. Moreover, students described issues such as pain, dryness, and discomfort during routine tasks like washing dishes or handling objects (Table 5). They also shared more emotional and practical burdens, including embarrassment when shaking hands, social self-consciousness, and routine changes. In our study, 8 (9.9%) students also stated that hand skin health influenced their career choices, with female students more often citing eczema or skin sensitivity to affect daily tasks or influence specialty choice (Table 5).

**Table 5:** Attitudes and perceptions towards hand skin health, skincare, and hygiene among clinical medical students.

**Question**	**Male** **No. (%)** **(*N* = 18)**	**Female** **No. (%)** **(*N* = 63)**	**Total** **No. (%)** **(*N* = 81)**	***p*-Value**
Perceived effectiveness at cleaning hands				
Soap	14 (77.8)	44 (69.8)	58 (71.6)	0.735
Hand sanitizer	0 (0)	1 (1.6)	1 (1.2)	
Both equally effective	4 (22.2)	18 (28.6)	22 (27.2)	
Perceived gentleness on skin				
Soap	14 (77.8)	44 (69.8)	58 (71.6)	0.735
Hand sanitizer	0 (0)	1 (1.6)	1 (1.2)	
Both equally gentle	4 (22.2)	18 (28.6)	22 (27.2)	
Perceived purpose of gloves in infection control				
To prevent infection from patient to student	0 (0)	1 (1.6)	1 (1.2)	0.857
To prevent infection from student to patient	1 (5.6)	4 (6.3)	5 (6.2)	
To prevent infection in both directions (student and patient)	17 (94.4)	58 (92.1)	75 (92.6)	
Who should provide hand lotions?				
Individual healthcare workers	2 (11.1)	4 (6.3)	6 (7.4)	0.720
Hospitals or clinics	5 (27.8)	22 (34.9)	27 (33.3)	
Both	11 (61.1)	37 (58.7)	48 (59.3)	
Hand skin health affecting future career decisions				
No	17 (94.4)	56 (88.9)	73 (90.1)	0.430
Yes	1 (5.6)	7 (11.1)	8 (9.9)	
Hand skin health affecting daily social life				
No	16 (88.9)	50 (79.4)	66 (81.5)	0.294
Yes	2 (11.1)	13 (20.6)	15 (18.5)	
*If yes, how? (free text)*	*13 free-text responses received, kindly view Supplementary material*			

N, number of participants; No., number; %, percentage.

## Discussion

The findings of this study draw important implications and raise awareness on hand skin health, the lack of which can have a larger impact than expected on future healthcare professionals. It also spotlights advocacy for local institutional change with regards to safeguarding hand skin health in all healthcare settings.

When comparing the hand hygiene practices and hand skin health between undergraduate medical students, there is a statistically significant increase in both the handwashing frequency and hand sanitizer use amongst clinical students in comparison to pre-clinical students. This can be explained by the increased clinical exposure, hands-on learning and infection control training, which is less emphasized during the pre-clinical years. This observed increase in hand hygiene frequency and associated worsening of hand skin health among clinical students is consistent with findings reported in other healthcare worker populations internationally, particularly following the COVID-19 pandemic. Studies from Europe and Asia have demonstrated similar associations between intensified infection control practices and increased prevalence of occupational hand dermatitis among healthcare workers and trainees. These parallels suggest that the patterns observed in our cohort likely reflect broader occupational trends rather than isolated institutional or temporal factors (6–8).

Additional exposure to hand hygiene products in this change of environment is also correlated with significantly worsened hand skin health among clinical students. Moreover, students in their clinical years are also statistically more likely to adhere to hand hygiene protocols due to directly interacting with patients and role-modeling the hand hygiene practices of senior healthcare staff.

One important factor to consider in infection control is the effectiveness of soap vs. hand-sanitiser (the latter is almost always alcohol-based gels and foams in healthcare settings). For instance, our study noted a widely-held belief that soap is more effective and gentler on hand skin than hand sanitiser. In actuality, the effectiveness of sanitiser vs. soap is variable (16, 17). Furthermore, sanitiser is gentler on hand skin, especially as moisturizing agents may be added by manufacturers (18). Misconception regarding the role of gloves as a substitute to hand hygiene practices in infection control was also present among some students, also reflective of bad practice in more senior healthcare professionals (19). The role of using gloves is to prevent transmission of infection from healthcare professional to patient and vice versa rather than eliminating infection itself (20, 21).

Poor hand skin health can act as a barrier to patient interaction and may influence interest in “high-contact” careers, particularly critical care and surgical specialties, as reported by 9.9% of students in this study. Although not statistically significant, poor skin health was also associated with reduced adherence to hand hygiene practices in 17.3% of students. These findings may contribute to the ongoing discussion that the art of physical examination is being lost among younger generations of healthcare students and professionals (22, 23). Previous literature has largely focused on changes in medical education and increasing reliance on diagnostic technologies as contributing factors, while occupational influences have received less attention. Chronic occupational hand dermatitis and hand eczema are known to impair daily hand function, with pain, fissuring, and irritation affecting routine hand use and work-related tasks (24). In clinical settings, physical examination requires frequent handwashing, sanitizer use, and prolonged glove wear, which may worsen symptoms in affected individuals (25). Persistent hand discomfort may therefore discourage repeated hands-on examination and tactile patient contact. In this context, hand skin health may represent a previously under-explored occupational factor contributing to changes in clinical examination practices and should be formally investigated on a larger scale.

This study is limited by its cross-sectional design and reliance on self-reported survey data, which may be subject to recall bias. In addition, the relatively small sample size and recruitment from a single medical school may limit the generalizability of the findings to other institutions or healthcare training environments. Despite these limitations, this study offers important insights into hand skin health among medical students in the United Arab Emirates, a population for which regional data are currently limited. As clinical year students are routinely exposed to infection control practices similar to those encountered in professional healthcare settings, these findings may help inform early preventive strategies. Previous studies have shown that investment in occupational hand skin health is ergonomic, cost-effective, and supports healthcare workers in maintaining patient contact and clinical performance (1–3), suggesting that similar measures may also benefit medical trainees. For instance, the implementation of lotion/cream stations nearby sinks and sanitizers in all local healthcare departments could improve the hand skin health of clinical students by encouraging preventative skincare behaviors. This would also play a hand in relieving distress caused by poor hand skin health reaching beyond the clinical environment, expressed by some students as “social embarrassment” when shaking hands and impairment in handling daily chores. Not only will these changes help clinical students, but widespread availability of these stations would also benefit healthcare workers and students at large. These interventions improve hand skin health and potentially hand hygiene as a consequence, as demonstrated in randomized controlled trials, such as by Filon et al. (3).

## Conclusion

This study underscores the importance of having preventative measures for occupational hand dermatitis in healthcare settings. As students transition into clinical training, they have increased exposure to infection control practices, which is associated with a deterioration in hand skin health. While increased handwashing and sanitiser use among clinical students reflects effective education and healthy practices, the associated worsening of skin health highlights an overlooked occupational concern which may affect student wellbeing and even career aspirations. Institutional investment in preventative strategies such as readily-available moisturizing stations is a practical and evidence-based step toward safeguarding skin integrity and prevent occupational hand dermatitis. Supporting hand skin health is an important component in protecting healthcare workers' wellbeing and sustaining safe infection control practices into their future careers.

## Data Availability

The original contributions presented in the study are included in the article/Supplementary material, further inquiries can be directed to the corresponding authors.
