# Supplementary material for: Tough on germs, gentle on hands: a cross-sectional study on hand skin health, skincare and hygiene habit changes among medical students in their clinical years in Dubai, the United Arab Emirates
Source: Front Public Health. 2026 Feb 13;13:1725837. doi: 10.3389/fpubh.2025.1725837 (PMC12946016; doi:10.3389/fpubh.2025.1725837)
Supplement: Supplementary file 1 [file Data_Sheet_1.pdf]

Supplementary material of the study titled: *Tough on Germs, Gentle on Hands: A Cross-Sectional Study on Hand Skin Health, Skincare, and Hygiene Habit Changes Among Medical Students in Their Clinical Years in Dubai, the United Arab Emirates*

Contents:

1. The study's questionnaire
2. Free-text responses to Question 43 (How does hand skin health affect your daily / social life?)

- A black bullet indicates single-answer questions
  - ○ A white bullet indicates a multiple-answer question

  [OPEN TEXT] indicates an open text option or question

Tough on germs, gentle on hands

Dear Student,

We need your help!

Please take 5-10 minutes to complete a short survey about hand skin conditions among MBRU medical students entering clinical environments. Your input is invaluable and will help us raise awareness and improve support for students like you.

In case you want to know more: this survey is conducted under the supervision of Dr. Volha Shpadaruk and Dr. Aida Azar. All responses are completely anonymous and confidential. Your participation is voluntary, and the results will only be used for research purposes.

By sharing your experiences, you'll play an important role in shaping how we address this issues for future medical students.

Continue below to start the survey and make a difference!

Section 1: Demographics & information

1. **Which cohort do you belong to?**
   - 2024
   - 2025
   - 2026

2. **What is your gender?**
   - Male
   - Female

3. **How old are you (in years)?**
   Please write your answer as a number e.g., "22"

4. **What is your nationality?**

   [OPEN TEXT]

5. **Do you have a family history of chronic skin diseases?**
    - No
    - Unsure
    - Eczema (atopic dermatitis)
    - Allergic contact dermatitis
    - Psoriasis
    - Other [OPEN TEXT]

6. **In the last 6 months spent during clinical rotations, have you worn nail varnish on most days (at least 3 days a week)?**
    - No
    - Yes, normal nail polish
    - Yes, gel polish
    - Yes, hard gel
    - Yes, acrylic nails

Section 2: Preclinical years

This section is dedicated to your hand hygiene and health during the preclinical years (1-3).

7. **During your preclinical years, did you have a history of any of the following chronic skin diseases?**
    - No
    - Unsure
    - Eczema (atopic dermatitis)
    - Allergic contact dermatitis
    - Irritant contact dermatitis
    - Psoriasis
    - Other [OPEN TEXT]

8. **Did any of the conditions you described in the previous question affect your hand skin? *This includes regions such as the palms, back of the hands, nails and extending to the wrist.***
    - No
    - Yes

9. **If you answered YES to the previous question, please select the symptoms that applied to your affected hand skin:**
    - Itching

- Burning / pain / tenderness
- Dryness / tightness
- Fissuring
- Blisters / vesicles
- Inability to perform daily activities
- Other [OPEN TEXT]

10. How many times have you seen a dermatologist or another doctor for your condition(s) during the preclinical years?
Please write your answer as a number e.g., "0" for never, "1" for once, "2" for twice, etc...

11. During your pre-clinical years, did you have a skincare routine for your hands? If so, what did it involve?
- No
- Yes - Prescription topical medication or ointments
- Yes - Non-prescription petroleum-containing cream (e.g., Vaseline)
- Yes - Non-prescription ceramide-containing cream
- Yes - Non-prescription urea-containing cream
- Yes - Non-prescription, unsure of main active ingredient
- Yes - Hand sanitizer
- Yes - Oral medication
- Other [OPEN TEXT]

12. During your pre-clinical years, how many times per day did you wash your hands with soap and water?
Please write your answer as a number e.g., "0" for never, "1" for once, "2" for twice, etc...

13. During your pre-clinical years, how many times per day did you use a hand sanitizer to clean your hands?
Please write your answer as a number e.g., "0" for never, "1" for once, "2" for twice, etc...

14. **During your pre-clinical years, how often did you clean your hands according to the protocol outlined in the image?**
On a scale of 1 to 5, where 1 = never, 5 = all the time

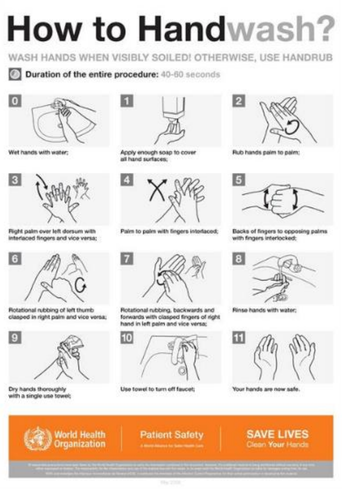

- 1
- 2
- 3
- 4
- 5

15. **During your preclinical years, according to the protocol in the image, approximately how much total time per day did you spend performing standard hand hygiene procedures?**

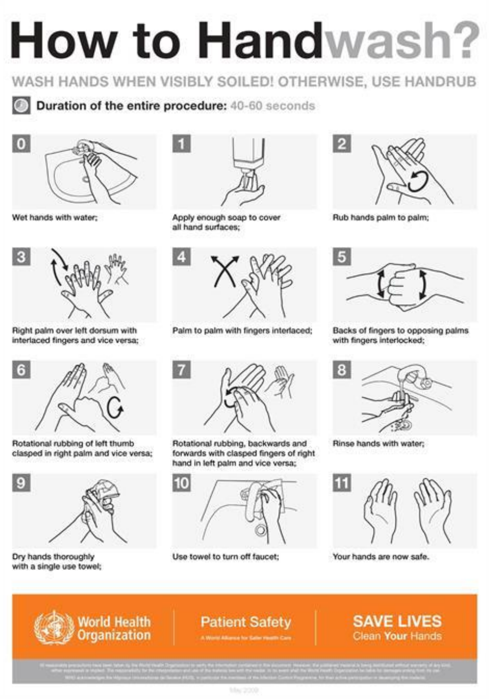

- Less than 5 minutes a day
- More than 5 minutes a day

Section 3: Clinical years

This section is dedicated to your hand hygiene and health during the clinical years (4-6).

16. **During your clinical years, did you notice any difference in your hand skin health in comparison to preclinical years?**
    - No difference
    - Yes - hand skin got better
    - Yes - hand skin got worse

17. **During your clinical years, did you have a history of any of the following chronic skin diseases?**
    - No
    - Unsure
    - Eczema (atopic dermatitis)
    - Allergic contact dermatitis
    - Irritant contact dermatitis
    - Psoriasis
    - Other [OPEN TEXT]

18. **Did any of the conditions you described in the previous question affect your hand skin? *This includes regions such as the palms, back of the hands, nails and extending to the wrist.***
    - No
    - Yes

19. **If you answered YES to the previous question, please select the symptoms that applied to your affected hand skin:**
    - Itching
    - Burning / pain / tenderness
    - Dryness / tightness
    - Fissuring
    - Blisters / vesicles
    - Inability to perform daily activities
    - Other [OPEN TEXT]

20. **How many times have you seen a dermatologist or another doctor for your condition(s) during your clinical years?**

Please write your answer as a number e.g., "0" for never, "1" for once, "2" for twice, etc...

21. Has entering the clinical environment altered how you care for your hand skin health?

- No
- Yes

22. During your clinical years, did you have a skincare routine for your hands? If so, what did it involve?

- o No
- o Yes - Prescription topical medication or ointments
- o Yes - Non-prescription petroleum-containing cream (e.g., Vaseline)
- o Yes - Non-prescription ceramide-containing cream
- o Yes - Non-prescription urea-containing cream
- o Yes - Non-prescription, unsure of main active ingredient
- o Yes - Hand sanitizer
- o Yes - Oral medication
- o Other [OPEN TEXT]

23. During your clinical years, how many times per day did you wash your hands with soap and water?
Please write your answer as a number e.g., "0" for never, "1" for once, "2" for twice, etc...

24. During your clinical years, how many times per day did you use a hand sanitizer to clean your hands?
Please write your answer as a number e.g., "0" for never, "1" for once, "2" for twice, etc...

25. During your clinical years, how often did you clean your hands according to the protocol outlined in the image?
On a scale of 1 to 5, where 1 = never, 5 = all the time

- 1
- 2
- 3
- 4
- 5

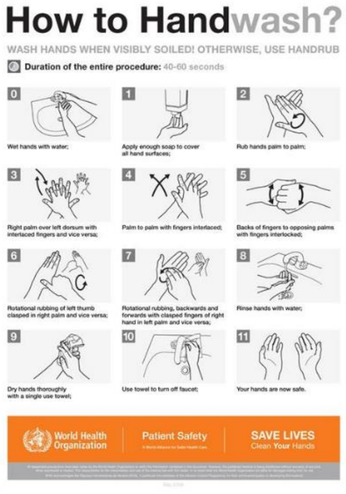

26. **During your clinical years, according to the protocol in the image, approximately how much total time per day did you spend performing standard hand hygiene procedures?**

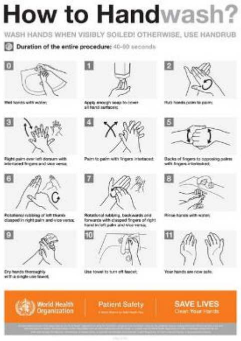

- Less than 5 minutes a day
- More than 5 minutes a day

27. Did your frequency of handwashing increase during specific clinical placements?

- No
- Yes

28. If you answered YES to the previous question, during which clinical placement(s)?

- Surgery (including sub-specialties and anesthesia)
- Medicine (including sub-specialities)
- Pediatrics
- Family Medicine
- Obstetrics and Gynecology
- Psychiatry
- Emergency medicine
- ICU/NICU
- Other [OPEN TEXT]

29. Did your skin health significantly worsen during specific clinical placement(s)?

- No
- Yes

30. If you answered YES to the previous question, during which clinical placement(s)?

- Surgery (including sub-specialties and anesthesia)
- Medicine (including sub-specialities)
- Pediatrics
- Family Medicine
- Obstetrics and Gynecology
- Psychiatry
- Emergency medicine
- ICU/NICU
- Other [OPEN TEXT]

31. During your clinical years, how many times per day did you use gloves?
Please write your answer as a number e.g., "0" for never, "1" for once, "2" for twice, etc...

[OPEN TEXT]

32. Do you have a latex allergy?

- No
- Yes

33. If you DO have a latex allergy, how accessible are latex-free gloves from your experience?
On a scale of 1 to 5, where 1 = never accessible, 5 = always accessible

- 1
- 2
- 3
- 4
- 5

34. In your personal experience, did experiencing poor hand skin health negatively affect your compliance with hand hygiene protocols?

- No
- Yes

35. If you answered YES to the previous question, please select how your compliance with hand hygiene protocols reduced in the clinical environment

- Wearing gloves to avoid handwashing
- Refraining from hands-on contact with patients
- Speeding through handwashing
- Not covering the entire hand whilst handwashing
- Avoiding using soap whilst handwashing
- Avoiding using hand sanitizer gel
- Other [OPEN TEXT]

Section 4: Additional questions

36. To the best of your knowledge, which is more effective at cleaning your hands: soap or hand sanitizer?

- Soap
- Hand sanitizer
- They are equally effective

37. To the best of your knowledge, which is gentler on the skin: soap or hand sanitizer?

- Soap
- Hand sanitizer
- They are equally gentle

38. To the best of your knowledge, how are gloves useful in terms of infection control?

- They prevent infection transmission from patients to medical students
- They prevent infection transmission from medical students to patients
- They do both of the above

39. In your opinion, should hand lotions be provided by hospitals / clinics; or is it the responsibility of individual healthcare workers?

- Individual healthcare workers
- Hospitals / clinics
- Both of the above

40. Does hand skin health affect your future career choice?

- No
- Yes

41. If you answered YES to the previous question, please describe how:

[OPEN TEXT]

42. Does hand skin health affect your daily / social life?

- No
- Yes

43. If you answered YES to the previous question, please describe how:

[OPEN TEXT]

Responses to Question 43: How does hand skin health affect your daily / social life?

13 respondents provided with answers to this optional question, as shown below:

Respondent #	Answer
1	Embarrassed to show hands due to excessive wrinkling, flare ups, difficulty washing hands/showering due to pain from hand lesions, inability to touch certain fabrics because hands stick to them, unexpected triggers to developing new rashes/unbearable itchiness, unable to interact with pets
2	I get more dry skin, which leads me to picking on the dry skin and leads to bleeding around my nail cuticles
3	It's annoying, makes me want to buy lotions to ensure I can use it with hand washing
4	Sometimes I take pictures of my coffee and like my hands to be pretty
5	Burning skin is bothersome, dry skin when hand shaking which can cause social embarrassment. Painful to perform some daily activities, things like opening a water bottle cap lacerates skin on palm of my hands because of the excessive dryness
6	The red, sometimes hyperpigmented, scaly skin on my hands makes me self conscious
7	Restricts my exposure to water that includes daily tasks such as washing dishes or wearing jewelry. Moreover, makes me to be more inclined in wearing gloves to reduce frequency of hand sanitizer use especially in clinics
8	Can't wash dishes efficiently or cut certain fruits like lemon
9	Trying to avoid touching germs so as to prevent regular hand washing
10	It would change my daily routines in terms of me including hand care routine in my daily life. Using creams more often
11	I developed very dry and cracked skin on my palms. I was especially ashamed when having to shake other people's hands as my hands were now rough to the touch
12	I like to have clean hands which are not dry because it makes me feel better when I work on my laptop, examine patients, when I play the piano, etc.
13	Longer time spent for skin care, time consumption to doctors appointments and treatment, cost of care, having to avoid triggers etc.
